# Cytotoxic Effects of Ardisiacrispin A from *Labisia pumila* on A549 Human Lung Cancer Cells

**DOI:** 10.3390/life14020276

**Published:** 2024-02-18

**Authors:** Yeong-Geun Lee, Tae Hyun Kim, Jeong Eun Kwon, Hyunggun Kim, Se Chan Kang

**Affiliations:** 1 Department of Oriental Medicine Biotechnology, College of Life Sciences, Kyung Hee University, Yongin 17104, Gyeonggi, Republic of Korea; lyg629@nate.com (Y.-G.L.); silsoo96@naver.com (T.H.K.); jjung@nmr.kr (J.E.K.); 2 Department of Biomechatronic Engineering, Sungkyunkwan University, Suwon 16419, Gyeonggi, Republic of Korea

**Keywords:** *Labisia pumila*, ardisiacrispin A, A549, natural product, triterpene

## Abstract

Background: Lung cancer is the predominant cause of cancer-related fatalities. This prompted our exploration into the anti-lung cancer efficacy of *Labisia pumila*, a species meticulously selected from the preliminary screening of 600 plants. Methods: Through the strategic implementation of activity-guided fractionation, ardisiacrispin A (1) was isolated utilizing sequential column chromatography. Structural characterization was achieved employing various spectroscopic methods, including nuclear magnetic resonance (NMR), mass spectrometry (MS), and infrared spectroscopy (IR). Results: *L. pumila* 70% EtOH extract showed significant toxicity in A549 lung cancer cells, with an IC_50_ value of 57.04 ± 10.28 µg/mL, as well as decreased expression of oncogenes and induced apoptosis. Compound **1**, ardisiacrispin A, induced a 50% cell death response in A549 cells at a concentration of 11.94 ± 1.14 µg/mL. Conclusions: The present study successfully investigated ardisiacrispin A extracted from *L. pumila* leaves, employing a comprehensive spectroscopic approach encompassing NMR, IR, and MS analyses. The anti-lung cancer efficacy of ardisiacrispin A and *L. pumila* extract was successfully demonstrated for the first time, to the best of our knowledge.

## 1. Introduction

Cancer obviously represents one of the biggest challenges to global human health [1]. Lung cancer is the predominant cause of cancer-related fatalities on a worldwide scale [2]. In 2020, lung cancers constituted 11.4% of newly diagnosed cancer cases, placing the lung as the second most prevalent site of incident cancers, and the majority of cases, approximately 85%, were attributed to a group of histological subtypes collectively known as non-small-cell lung cancer [2,3]. Epidermal growth factor receptor (EGFR) tyrosine kinase inhibitors such as gefitinib and erlotinib are typically the first chemotherapy treatments for lung cancer. Unfortunately, the prognosis of advanced and recurrent lung cancer remains suboptimal, and standard treatments utilizing cytotoxic anticancer drugs demonstrate limited therapeutic effectiveness [4]. In the current landscape of cancer management, diverse treatment options such as chemotherapy, surgery, and radiotherapy exist [5,6]. Despite their efficacy, these therapeutic interventions are often associated with severe side effects, posing a substantial risk to patients, or imposing exacting prerequisites for their implementation.

Given the imperative to advance anti-lung cancer drug development, we conducted a screening of potential candidates from a repository of plants within our institute (data not presented). *Labisia pumila* (Myrsinaceae), grown in southeast Asia, emerged as a promising anti-lung cancer candidate in our preliminary investigation [7]. Traditionally employed for the maintenance of female reproductive health and postpartum care, this botanical specimen holds promise in anti-cancer applications. The investigation of this plant has revealed a number of secondary metabolites, exhibiting phytoestrogenic, anti-bacterial, anti-fungal, anti-oxidant, anti-carcinogenic, and anti-aging effects [7,8,9]. Historical uses of this botanical entity include the enhancement of stamina and treatment for various conditions, such as dysentery, rheumatism, gonorrhea, excessive flatulence, and cancers, particularly those affecting the breast and uterus [10,11]. Despite the well-established anti-cancer properties of *L. pumila*, limited information is available concerning its efficacy in the context of lung cancer. Therefore, in the present study, the natural product, *L. pumila*, and its active compound were rigorously investigated to assess their potential as a candidate for lung cancer treatment.

## 2. Materials and Methods

### 2.1. Reagents and Instrumentation

The equipment and chemicals for isolation and structural elucidation of the anti-cancer component were referred to in our previous investigations [11,12]. Briefly, SiO_2_ (Kieselgel 60, Merck, Darmstadt, Germany) and ODS (Lichroprep RP-18, 40–60 μm, Merck) were used as resins for column chromatography (c.c.). The separated compound was detected using a UV lamp (Spectroline Model ENF-240 C/F, Spectronics Corporation, Westbury, NY, USA) following application on Kieselgel 60 F254 (Merck) and Kieselgel RP-18 F254S (Merck) plates, subsequent to spraying with a 10% aqueous H_2_SO_4_ solution. Nuclear magnetic resonance (NMR) spectra were recorded employing a Bruker Avance 600 (Billerica, MA, USA), and melting points were precisely determined using a Fisher-John Melting Point Apparatus (Fisher Scientific, Miami, FL, USA). Deuterium solvents for measurement of NMR and standard organic solvents for extraction were purchased from Sigma Aldrich Co., Ltd. (St. Louis, MO, USA) and Daejung Chemical Ltd. (Seoul, Republic of Korea), respectively.

Tryptic Soy Broth (TSB) was purchased from KisanBio (Seoul, Republic of Korea). Doxorubicin, dimethyl sulfoxide (DMSO), and MTT [3-(4,53-(4,5-dimethylthiazol-2-yl)-2,5-diphenyltetrazolium bromide)] were purchased from Sigma-Aldrich (St. Louis, MO, USA). Dulbecco’s modification of Eagle’s media (DMEM) with 4.5 g/L glucose, L-glutamine, and sodium pyruvate, and RPMI1640 with L-glutamine were purchased from Mediatech (Manassas, VA, USA). Fetal bovine serum (FBS), bovine calf serum (BCS), trypsin-EDTA, and penicillin–streptomycin were purchased from Thermo Fisher Scientific (Waltham, MA, USA).

### 2.2. Plants

In this study, a comprehensive set of 600 plant extract samples was sourced from the International Biological Material Research Center (IBMRC, Cheongju, Republic of Korea). Voucher specimens, uniquely identified by the codes KHU-BMRI-2017-001 through KHU-BMRI-2017-600, were deposited at the Bio-Medical Research Institute, Yongin, Kyung Hee University. The process for isolating potential anti-cancer candidates involved the extraction of plant materials using 100% methanol (MeOH), and the extracts were subsequently solubilized to a concentration of 10 mg/mL in dimethylsulfoxide (DMSO). 

### 2.3. Isolation of the Anti-Cancer Component from L. pumila

Dried leaves of *L. pumila* (180 g), purchased from Malaysia, were chopped into small pieces and subjected to extraction using 70% aqueous EtOH (3 L × 3) for 24 h at room temperature. The EtOH extract (11 g), obtained through filtration and subsequent concentration in vacuo, was reconstituted in 200 mL of H_2_O. Subsequent sequential extractions were performed three times using *n*-hexane (150 mL), dichloromethane (DCM, 150 mL), ethyl acetate (EtOAc, 150 mL), and *n*-BuOH (150 mL), resulting in distinct fractions: *n*-hexane (LPH, 2.44 g), DCM (LPD, 1.07 g), EtOAc (LPE, 390 mg), *n*-BuOH (LPB, 930 mg), and aqueous (LPA, 6.17 g) fractions. Among the fractions obtained, LPD (1.07 g), identified through activity-guided fractionation, underwent further fractionation using SiO_2_ cc (Ø 4 × 15 cm, CHCl_3_:MeOH:H_2_O = 50:3:1→30:3:1→20:3:1→10:3:1→7:3:1→5:3:1, 1 L of each), resulting in the isolation of 21 fractions (LPD-1 to LPD-21). Fraction LPD-13 (82.0 mg, elution volume/total volume (V_e_/V_t_) 0.644–0.751) underwent additional fractionation employing ODS cc (Ø 1 × 5 cm, acetone: H_2_O = 1:3 → 3:1, 150 mL of each), yielding 6 fractions (LPD-13-1 to LPD-13-6). Ardisiacrispin A (1) was successfully isolated from LPD-13-3 (40.0 mg) using TLC (SiO_2_) with elution in CHCl_3_: MeOH: H_2_O (5:3:1) within the range of 0.160–0.220 and TLC (ODS) with acetone:H_2_O (1:1) within the range of 0.270–0.360.

Ardisiacrispin A (1): white amorphous powder; negative FAB/MS *m*/*z* 1059 [M-H]^−^; negative HR-FAB/MS *m*/*z* 1059.5375 [M−H]^−^(calculated for C_52_H_83_O_22_, 1059.5376); melting point: 229–230 °C; IR (KBr, ν_max_, cm^−1^): 3415, 3455, 3570 (OH), 1710 (CHO); ^1^H-NMR (600 MHz, DMSO-*d*_6_, *δ*_H_, *J* in Hz) 9.61 (1H, s, H-30), 5.36 (1H, d, *J* = 7.2 Hz, H-glc′-1), 4.97 (1H, d, *J* = 7.8 Hz, H-glc″-1), 4.96 (1H, d, *J* = 7.2 Hz, H-xyl-1), 4.76 (1H, d, *J* = 5.2 Hz, H-ara-1), 4.58 (1H, br.d, *J* = 9.6 Hz, H-ara-5a), 4.48 (1H, br.dd, *J* = 11.4, 8.4 Hz, H-glc′-6a), 4.44 (1H, ddd, *J* = 10.4, 7.8 Hz, H-xyl-5a), 4.44 (1H, dd, *J* = 9.6, 5.2 Hz, H-ara-2), 4.39 (1H, br.dd, *J* = 11.4, 8.4 Hz, H-glc″-6a), 4.31 (1H, ddd, *J* = 11.4, 8.4, 4.8 Hz, H-glc’-6b), 4.26 (1H, ddd, *J* = 11.4, 8.4, 4.8 Hz, H-glc″-6b), 4.23 (1H, m, H-ara-4), 4.22 (1H, dd, *J* = 7.8, 7.8 Hz, H-xyl-3), 4.21 (1H, m, H-16), 4.20 (1H, dd, *J* = 7.8, 7.8 Hz, H-glc″-3), 4.17 (1H, m, H-xyl-4), 4.13 (1H, dd, *J* = 8.4, 7.2 Hz, H-glc′-4), 4.11 (1H, dd, *J* = 9.6, 9.6 Hz, H-ara-3), 4.11 (1H, dd, *J* = 8.4, 7.8 Hz, H-glc″-4), 4.02 (1H, dd, *J* = 7.8, 7.2 Hz, H-glc′-2), 3.96 (1H, dd, *J* = 7.2, 7.2 Hz, H-glc′-3), 3.96 (1H, br.d, *J* = 8.4 Hz, H-glc′-5), 3.93 (1H, dd, *J* = 7.2, 7.8 Hz, H-xyl-2), 3.83 (1H, br.d, *J* = 8.4 Hz, H-glc″-5), 3.78 (1H, dd, *J* = 7.8, 7.8 Hz, H-glc″-2), 3.77 (1H, br.d, *J* = 9.6 Hz, H-ara-5b), 3.52 (1H, ddd, *J* = 10.4, 4.2 Hz, H-xyl-5b), 3.51 (1H, d, *J* = 7.8 Hz, H-28a), 3.16 (1H, dd, *J* = 12.0, 4.2 Hz, H-3), 3.16 (1H, d, *J* = 7.8 Hz, H-28b), 2.78 (1H, dd, *J* = 14.4, 4.2 Hz, H-19a), 2.50 (2H, dd, *J* = 13.2, 4.2 Hz, H-21), 2.14 (1H, br.d, *J* = 10.8 Hz, H-15a), 2.10 (1H, t, *J* = 7.2 Hz, H-12a), 2.06 (1H, br.d, *J* = 14.4 Hz, H-19b), 1.99 (1H, overlapped, H-22a), 1.96 (1H, br.d, *J* = 12.0 Hz, H-2a), 1.79 (2H, overlapped, H-11), 1.79 (1H, dd, *J* = 12.0, 4.2 Hz, H-2b), 1.60 (1H, br.d, *J* = 12.6 Hz, H-1a), 1.53 (1H, overlapped, H-22b), 1.50 (3H, s, H-27), 1.50 (1H, overlapped, H-15b), 1.40 (1H, overlapped, H-12b), 1.37 (1H, overlapped, H-6a), 1.36 (1H, dd, *J* = 14.4, 4.2 Hz, H-18), 1.26 (3H, s, H-26), 1.22 (1H, overlapped, H-6b), 1.20 (1H, overlapped, H-9), 1.18 (2H, overlapped, H-7), 1.17 (3H, s, H-23), 1.03 (3H, s, H-24), 1.00 (3H, s, H-29), 0.81 (3H, s, H-25), 0.81 (1H, br.d, *J* = 12.6 Hz, H-1b), 0.65 (1H, d, *J* = 10.8 Hz, H-5); ^13^C-NMR (150 MHz, DMSO-*d*_6_, *δ*_C_) 207.4 (C-30), 105.7 (C-xyl-1), 104.1 (C-glc′-1), 104.0 (C-ara-1), 102.9 (C-glc″-1), 88.3 (C-3), 85.6 (C-13), 83.7 (C-glc″-2), 79.2 (C-ara-2), 78.1 (C-ara-4), 77.1 (C-glc″-5), 76.9 (C-glc′-5), 76.8 (C-glc′-3), 76.2 (C-glc″-3), 76.1 (C-16), 76.0 (C-28), 75.9 (C-xyl-3), 75.1 (C-glc′-2), 74.3 (C-xyl-2), 71.7 (C-xyl-4), 70.8 (C-ara-3), 70.7 (C-glc′-4), 70.2 (C-glc″-4), 64.2 (C-ara-5), 62.8 (C-xyl-5), 61.3 (C-glc″-6), 61.1 (C-glc′-6), 54.9 (C-5), 52.6 (C-18), 49.6 (C-9), 47.6 (C-20), 43.8 (C-14), 43.1 (C-17), 41.7 (C-8), 38.9 (C-4), 38.5 (C-1), 36.1 (C-10), 35.7 (C-15), 33.8 (C-7), 32.6 (C-19), 31.7 (C-22), 31.4 (C-12), 29.7 (C-21), 27.4 (C-23), 25.9 (C-2), 23.4 (C-29), 19.1 (C-27), 18.7 (C-11), 18.1 (C-26), 17.3 (C-6), 15.7 (C-24), 15.3 (C-25).

### 2.4. Cell Viability and Cytotoxicity Assay

A549 human cell lines, obtained from the Korean Cell Line Bank (KCLB), were cultured in RPMI1640 supplemented with 10% fetal bovine serum (FBS) and 1% penicillin/streptomycin [13]. The cells were cultivated in a humidified incubator at 37 °C with a CO_2_ concentration of 5%. Cell viability was evaluated utilizing the MTT assay, with seeding densities of 5 × 10^3^ cells/well in a 96-well plate. Following a 24 h incubation period, the culture medium was replaced, and the samples were subjected to experimental treatments. After 24 h, the cells were stained using MTT solution in PBS, resulting in a final concentration of 0.5 mg/mL. The cells were subjected to a 4 h incubation at 37 °C. Upon completion of this incubation period, the supernatant was removed and 100 µL of DMSO was added. Utilizing a microplate reader (Tecan, Switzerland), absorbance was measured at 540 nm. The cell cytotoxicity rates were calculated based on the optical density readings, expressed as percentages relative to the vehicle control, and this procedure was repeated for accuracy.

### 2.5. Tali^TM^ Cell Cycle Assay

Human A549 cells were seeded in 6-well plates at a density of 1 × 10^5^ cells/well. After 24 h incubation, the culture medium was replaced, and the cells were subjected to specific treatments. Following fixation, the cells were treated with the optimized Tali^TM^ cell cycle reagent (Thermo, Middlesex, MA, USA) and incubated in darkness for 30 min. The Tali^®^ image cytometer (Thermo, Middlesex, MA, USA) was employed for cell cycle analysis. The acquired cell cycle data from the Tali^TM^ image-based cytometer were analyzed both on the instrument and through dedicated cell cycle modeling software.

### 2.6. Statistical Analysis

All data are presented as mean ± standard error of the mean (SEM). The significance of differences between groups was assessed using one-way analysis of variance (ANOVA). Statistical significance was defined as *p* < 0.05.

## 3. Results and Discussion

### 3.1. Determination of the Anti-Cancer Agent and Its Optimal Extraction Condition

In pursuit of identifying a natural anti-cancer candidate agent, a pilot study was conducted through cell viability assays to determine IC_50_ values in A549 cells. From an extensive pool of MeOH extracts obtained from a diverse collection of over 600 plants, *L. pumila* emerged as a potent anti-lung cancer candidate. Following this selection, a systematic evaluation was carried out to establish the optimal concentration of the *L. pumila* extract. The evaluation revealed the significant toxicity demonstrated by the 70% EtOH extract in A549 lung cancer cells, with an IC_50_ value of 57.04 ± 10.28 µg/mL (Table 1).

### 3.2. Structural Evaluation of the Anti-Lung Cancer Component from L. pumila

The dried leaves of *L. pumila* were subjected to extraction using 70% aqueous EtOH, and the resulting concentrate was fractioned into *n*-hexane (LPH), dichloromethane (LPD), ethyl acetate (LPE), *n*-BuOH (LPB), and H_2_O (LPW) fractions. A series of activity-guided fractionation steps, employing SiO_2_, ODS, and Sephadex LH-20 column chromatography (c.c.) for the LPD fraction, led to the isolation of a singular triterpenoid saponin (1). Elucidation of its chemical structure was achieved through a comprehensive analysis of spectroscopic data, including mass spectrometry (MS), infrared spectroscopy (IR), and NMR (both 1D and 2D).

Compound **1**, a white amorphous powder (MeOH), showed IR absorbance bands of hydroxyl (3415, 3455, and 3570 cm^−1^) and formyl groups (1710 cm^−1^). The molecular formula of Compound **1** was determined to be C_52_H_83_O_22_ through negative fast atom bombardment mass spectrometry (FAB/MS) *m*/*z* 1059 [M − H]^−^ and negative high-resolution FAB/MS *m*/*z* 1059.5375 [M − H]^−^ (calcd for C_52_H_83_O_22_, 1059.5376). The ^1^H-NMR spectrum (600 MHz, DMSO-d_6_) showed proton signals due to six singlet methyls [δ_H_ 1.50 (3H, s, H-27), δ_H_ 1.26 (3H, s, H-26), δ_H_ 1.17 (3H, s, H-23), δ_H_ 1.03 (3H, s, H-24), δ_H_ 1.00 (3H, s, H-29), and δ_H_ 0.81 (3H, s, H-25)]; one formyl [δ_H_ 9.61 (1H, s, H-30)]; two oxygenated methines [δ_H_ 4.21 (1H, m, H-16) and δ_H_ 3.16 (1H, dd, *J* = 12.0, 4.2 Hz, H-3)]; two oxygenated methylenes [δ_H_ 3.51 (1H, d, *J* = 7.8 Hz, H-28a) and δ_H_ 3.16 (1H, d, *J* = 7.8 Hz, H-28b)]; three methines [δ_H_ 1.36 (1H, dd, *J* = 14.4, 4.2 Hz, H-18), δ_H_ 1.20 (1H, overlapped, H-9), and δ_H_ 0.65 (1H, d, *J* = 10.8 Hz, H-5)]; and ten methylenes [δ_H_ 2.78 (1H, dd, *J* = 14.4, 4.2 Hz, H-19a), δ_H_ 2.50 (2H, dd, *J* = 13.2, 4.2 Hz, H-21), δ_H_ 2.14 (1H, br.d, *J* = 10.8 Hz, H-15a), δ_H_ 2.10 (1H, t, *J* = 7.2 Hz, H-12a), δ_H_ 2.06 (1H, br.d, *J* = 14.4 Hz, H-19b), δ_H_ 1.99 (1H, overlapped, H-22a), δ_H_ 1.96 (1H, br.d, *J* = 12.0 Hz, H-2a), δ_H_ 1.79 (2H, overlapped, H-11), δ_H_ 1.79 (1H, dd, *J* = 12.0, 4.2 Hz, H-2b), δ_H_ 1.60 (1H, br.d, *J* = 12.6 Hz, H-1**a**), δ_H_ 1.53 (1H, overlapped, H-22b), δ_H_ 1.50 (1H, overlapped, H-15b), δ_H_ 1.40 (1H, overlapped, H-12b), δ_H_ 1.37 (1H, overlapped, H-6a), δ_H_ 1.22 (1H, overlapped, H-6b), δ_H_ 1.18 (2H, overlapped, H-7), and δ_H_ 0.81 (1H, br.d, *J* = 12.6 Hz, H-1b)]. The proton signals indicated the aglycone of Compound **1** to be an oleanane-type triterpenoid possessing a formyl group. Also, four hemiacetals [δ_H_ 5.36 (1H, d, *J* = 7.2 Hz, H-glc′-1), δ_H_ 4.97 (1H, d, *J* = 7.8 Hz, H-glc″-1), δ_H_ 4.96 (1H, d, *J* = 7.2 Hz, H-xyl-1), δ_H_ 4.76 (1H, d, *J* = 5.2 Hz, H-ara-1)]; fourteen oxygenated methines [δ_H_ 4.44 (1H, dd, *J* = 9.6, 5.2 Hz, H-ara-2), δ_H_ 4.23 (1H, m, H-ara-4), δ_H_ 4.22 (1H, dd, *J* = 7.8, 7.8 Hz, H-xyl-3), δ_H_ 4.20 (1H, dd, *J* = 7.8, 7.8 Hz, H-glc″-3), δ_H_ 4.17 (1H, m, H-xyl-4), δ_H_ 4.13 (1H, dd, *J* = 8.4, 7.2 Hz, H-glc′-4), δ_H_ 4.11 (1H, dd, *J* = 9.6, 9.6 Hz, H-ara-3), δ_H_ 4.11 (1H, dd, *J* = 8.4, 7.8 Hz, H-glc″-4), δ_H_ 4.02 (1H, dd, *J* = 7.8, 7.2 Hz, H-glc′-2), δ_H_ 3.96 (1H, dd, *J* = 7.2, 7.2 Hz, H-glc′-3), δ_H_ 3.96 (1H, br.d, *J* = 8.4 Hz, H-glc′-5), δ_H_ 3.93 (1H, dd, *J* = 7.2, 7.8 Hz, H-xyl-2), δ_H_ 3.83 (1H, br.d, *J* = 8.4 Hz, H-glc″-5), and δ_H_ 3.78 (1H, dd, *J* = 7.8, 7.8 Hz, H-glc″-2)]; and four germinal oxygenated methylene proton [δ_H_ 4.58 (1H, br.d, *J* = 9.6 Hz, H-ara-5a), δ_H_ 4.48 (1H, br.dd, *J* = 11.4, 8.4 Hz, H-glc′-6a), δ_H_ 4.44 (1H, ddd, *J* = 10.4, 7.8 Hz, H-xyl-5a), δ_H_ 4.39 (1H, br.dd, *J* = 11.4, 8.4 Hz, H-glc″-6a), δ_H_ 4.31 (1H, ddd, *J* = 11.4, 8.4, 4.8 Hz, H-glc′-6b), δ_H_ 4.26 (1H, ddd, *J* = 11.4, 8.4, 4.8 Hz, H-glc″-6b), δ_H_ 3.77 (1H, br.d, *J* = 9.6 Hz, H-ara-5b), δ_H_ 3.52 (1H, ddd, *J* = 10.4, 4.2 Hz, H-xyl-5b)] signals were observed as the proton signals of four hexoses. The coupling constants of the anomer proton signals of three sugars (glc′-1, glc-″-1, xyl-1; *J* = 7.8 or 7.2 Hz) and one sugar (ara-1; *J* = 5.2 Hz) confirmed the axial–axial and axial–equatorial configurations of the anomer hydroxyl groups, respectively.

The ^13^C-NMR data exhibited a total of 30 carbon signals corresponding to the aglycone along with 22 carbons derived from four hexoses, indicating Compound **1** to be a triterpenoid with four hexoses. The ^13^C-NMR (150 MHz, DMSO-d_6_) spectrum showed one formyl carbon signal, δ_C_ 207.4 (C-30); one oxygenated quaternary, δ_C_ 85.6 (C-13); one oxygenated methylene, δ_C_ 76.0 (C-28); two oxygenated methines [δ_C_ 88.3 (C-3) and δ_C_ 76.1 (C-16)]; six quaternaries [δ_C_ 47.6 (C-20), δ_C_ 43.8 (C-14), δ_C_ 43.1 (C-17), δ_C_ 41.7 (C-8), δ_C_ 38.9 (C-4), and δ_C_ 36.1 (C-10)]; three methines [δ_C_ 54.9 (C-5), δ_C_ 52.6 (C-18), and δ_C_ 49.6 (C-9)]; ten methylenes [δ_C_ 38.5 (C-1), δ_C_ 35.7 (C-15), δ_C_ 33.8 (C-7), δ_C_ 32.6 (C-19), δ_C_ 31.7 (C-22), δ_C_ 31.4 (C-12), δ_C_ 29.7 (C-21), δ_C_ 25.9 (C-2), δ_C_ 18.7 (C-11), and δ_C_ 17.3 (C-6)]; and six methyls [δ_C_ 27.4 (C-23), δ_C_ 23.4 (C-29), δ_C_ 19.1 (C-27), δ_C_ 18.1 (C-26), δ_C_ 15.7 (C-24), and δ_C_ 15.3 (C-25)]. Based on the chemical shifts of the sugar carbon signals, we observed four hemiacetals [δ_C_ 105.7 (C-xyl-1), δ_C_ 104.1 (C-glc′-1), δ_C_ 104.0 (C-ara-1), and δ_C_ 102.9 (C-glc″-1)]; fourteen oxygenated methines [δ_C_ 83.7 (C-glc″-2), δ_C_ 79.2 (C-ara-2), δ_C_ 78.1 (C-ara-4), δ_C_ 77.1 (C-glc″-5), δ_C_ 76.9 (C-glc′-5), δ_C_ 76.8 (C-glc’-3), δ_C_ 76.2 (C-glc″-3), δ_C_ 75.9 (C-xyl-3), δ_C_ 75.1 (C-glc′-2), δ_C_ 74.3 (C-xyl-2), δ_C_ 71.7 (C-xyl-4), δ_C_ 70.8 (C-ara-3), δ_C_ 70.7 (C-glc′-4), and δ_C_ 70.2 (C-glc″-4)]; and four oxygenated methylenes [δ_C_ 64.2 (C-ara-5), δ_C_ 62.8 (C-xyl-5), δ_C_ 61.3 (C-glc″-6), and δ_C_ 61.1 (C-glc′-6)]; these sugars were determined to be two β-glucopyranoses, one α-xylopyranose, and one α-arabinopyranose, respectively. The oxygenated methine resonances of aglycone (C-3), glucose (C-glc″-2), and arabinose (C-ara-2 and C-ara-4) were detected at lower magnetic fields (δ_C_ 88.3, δ_C_ 83.7, δ_C_ 79.2, and δ_C_ 78.1) than the commonly detected chemical shift (δ_C_ 78, δ_C_ 75, δ_C_ 71, and δ_C_ 71). This discrepancy, attributed to glycosidation-induced shifts, provided conclusive evidence confirming the precise positions of the glycosidic linkage.

**Figure 1:**
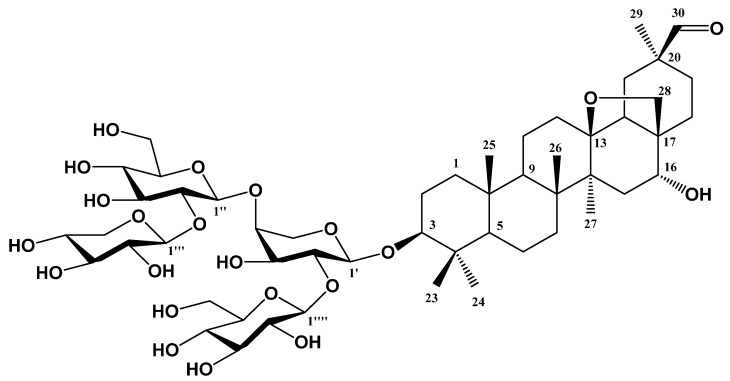
Chemical structure of ardisiacrispin A (**1**) extracted from *L. pumila* leaves.

In the gHMBC spectrum, one formyl [δ_H_ 9.61 (1H, s, H-30)] proton signal showed cross-peaks with the quaternary carbon signal δ_C_ 47.6 (C-20), and oxygenated methylenes [δ_H_ 3.51 (1H, d, *J* = 7.8 Hz, H-28a) and δ_H_ 3.16 (1H, d, *J* = 7.8 Hz, H-28b)] showed cross-peaks with the oxygenated quaternary carbon δ_C_ 85.6 (C-13) and oxygenated methine carbon δ_C_ 76.1 (C-16) signals. Furthermore, the four anomer proton signals of two glucoses [δ_H_ 5.36 (1H, d, *J* = 7.2 Hz, H-glc′-1) and δ_H_ 4.97 (1H, d, *J* = 7.8 Hz, H-glc″-1)], one xylose [δ_H_ 4.96 (1H, d, *J* = 7.2 Hz, H-xyl-1)], and one arabinose [δ_H_ 4.76 (1H, d, *J* = 5.2 Hz, H-ara-1)] showed cross-peaks with four oxygenated methine carbon signals [δ_C_ 88.3 (C-3), δ_C_ 83.7 (C-glc″-2), δ_C_ 79.2 (C-ara-2), and δ_C_ 78.1 (C-ara-4)]. This spectral evidence strongly suggests the positioning of arabinose at the C-3 of the aglycone, xylose at the C-glc″-2 of the glucopyranose moiety, and the two glucoses at the C-ara-2 and C-ara-4 of the arabinose moiety, respectively. Taken together, ardisiacrispin A (3*β*-*O*-[*α*-L-xylopyranosyl-(1→2)-*O*-*β*-D-glucopyranosyl-(1→4)-[*O*-*β*-D-glucopyranosyl-(1→2)]-*α*-L-arabinopyranosyl]-16*α*-hydroxy-13*β*,28-epoxyolean-30-al; **1**) was identified as the chemical structure of Compound **1** (Figure 1). Another study also investigated the spectroscopic parameters of Compound **1** [14]. Compound **1** was isolated from the *L. pumila* leaves for the first time in this study. Originally documented in 1987 from *Ardisia crispa*, Compound **1** has been recognized for its cytotoxic efficacy against diverse cancer cell lines, including NCI-H46; SF-268; MCF-7; melanoma WM793, HTB140, and A375 (skin panel); prostate cancer Du145 and PC3 and normal prostate epithelial PNT2 (prostate panel); colon cancer Caco2 and HT29; and HepG2 (gastrointestinal panel) liver cells [15,16,17,18].

### 3.3. Regulation of Cell Cycle by L. pumila

Apoptosis, an intricate mechanism of programmed cell death inherent to multicellular organisms, serves as a pivotal process for the elimination of undesirable and defective cells [19]. This orchestrated cellular death not only facilitates the removal of superfluous entities but also mitigates the risk of inciting undesirable inflammatory responses. Apoptosis is a ubiquitous phenomenon, actively participating during normal development and cellular turnover, as well as extending to various pathological conditions.

The cell cycle, a highly conserved mechanism, orchestrates the replication of eukaryotic cells. The regulation of cell death is intricately linked to genes governing cell cycle progression. Cumulative evidence has underscored the impact of cell cycle manipulation on modulating apoptosis reactions, contingent upon the specific cellular context [20,21]. 

**Figure 2:**
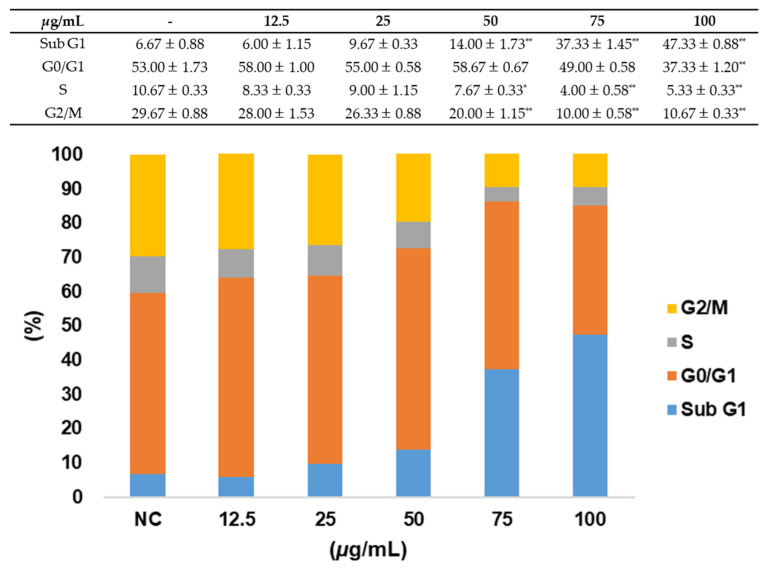
Regulation of cell cycle by *L. pumila* extract. A549 cells were treated with varying concentrations (0–100 μg/mL) of *L. pumila* extract for 24 h, and the effects were assessed using a Tali^TM^ image-based cytometer. * *p* < 0.05, ** *p* < 0.01.

Concentration-dependent reductions in the G0/G1, G2/M, and S phases were discerned in response to the *L. pumila* extract. Conversely, an elevation in the Sub G1 phase was observed upon treatment with the *L. pumila* extract. It is well documented that an augmentation in the Sub G1 phase corresponds to the onset of apoptosis. Thus, the observed elevation in the Sub G1 phase following treatment with the *L. pumila* extract can be attributed to the induction of apoptosis (Figure 2).

### 3.4. Evaluation of the Anti-Lung Cancer Efficacy of L. pumila and Ardisiacrispin A

**Figure 3:**
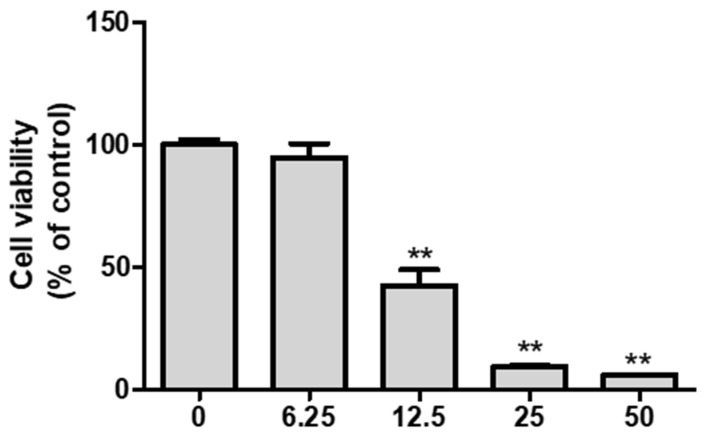
Cytotoxicity against A549 cells of ardisiacrispin A (**1**) from *L. pumila* leaves. Data are presented as the mean ± standard deviation. *n* = 3, ** *p* < 0.01.

Compound **1** induced a 50% cell death response in A549 cells at a concentration of 11.94 ± 1.14 µg/mL (Figure 3). Natural products are inexpensive and relatively safe compared with synthetics as extensive research studies have been conducted on the development of anti-cancer candidates for decades [22,23]. In particular, compared with dehydrocostuslactone (15 μg/mL) from *Aucklandia lappa*; resveratrol (2.04 ± 0.3 µg/mL) from grape; isochaihulactone (7.37 ± 2.03 µg/mL) from *Bupleurum scorzonerifolium*, ixerinoside (29 µg/mL), and ixerin Z (25 µg/mL); and 3-hydroxydehydroleucodin (15 µg/mL) from *Ixeris sonchifolia*, the compound we investigated in this study, ardisiacrispin A, revealed a significant inhibition efficacy (11.94 ± 1.14 µg/mL) against A549 cells [23,24,25,26]. Although this compound has a lower inhibition effect than the synthetics vincristine (1.81 ± 0.25 ng/mL) and paclitaxel (2.22 ± 0.43 ng/mL), *L. pumila* and ardisiacrispin A are anti-lung cancer candidates thanks to their advantage of having no or minor adverse effects [26].

**Figure 4:**
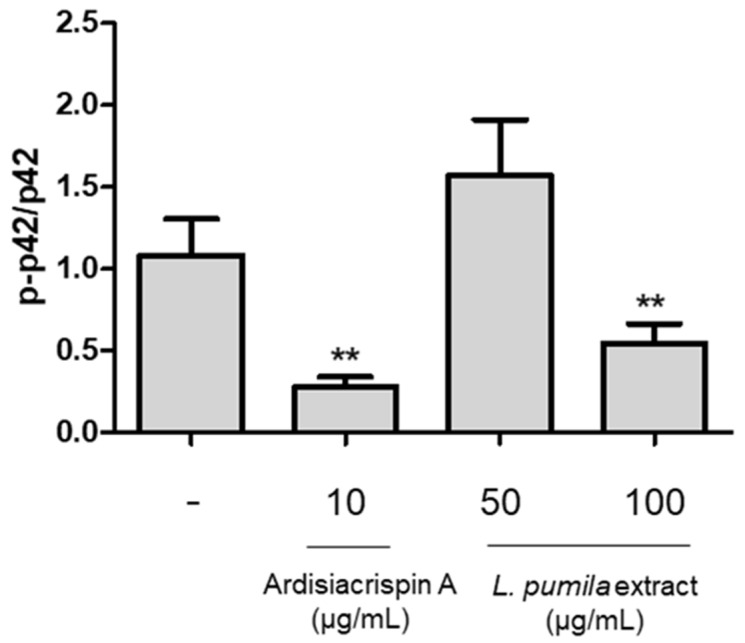
Changes in protein expression levels (p-ERK and ERK) associated with cell cycle regulation. Data are presented as the mean ± standard deviation. *n* = 3, ** *p* < 0.01.

Extracellular signal-regulated kinase (ERK) plays a crucial role in tumorigenesis [27]. ERK activity is associated with the promotion of apoptotic pathways, including the induction of mitochondrial cytochrome C release, caspase-8 activation, permanent cell cycle arrest, and autophagic vacuolization. The active state of ERK is characterized by its phosphorylated form [28,29,30]. Figure 4 demonstrates that the p-ERK/ERK ratio in ardisiacrispin A-treated cells was lower than the normal control. In addition, *L. pumila* revealed a dose-dependent decrease in the p-ERK/ERK ratio.

## 4. Conclusions

The present study successfully extracted ardisiacrispin A (**1**) from *L. pumila* leaves, employing a comprehensive spectroscopic approach encompassing NMR, IR, and MS analyses. Compound **1** was isolated from *L. pumila* leaves for the first time in this study. *L. pumila* and its active compound, ardisiacrispin A (**1**), demonstrated potential in suppressing the proliferation and metastasis of lung cancer cells. This inhibitory effect is attributed to the modulation of oncogenic signaling pathways related to EGFR and FGER in lung cancer. Although further apoptosis studies are needed to investigate the involved apoptotic pathways, this is the first report to demonstrate the anti-lung cancer efficacy of ardisiacrispin A (**1**) and *L. pumila* extract, to the best of our knowledge. Consequently, these findings underscore the feasibility of utilizing ardisiacrispin A (**1**) and *L. pumila* extract as anti-lung cancer agents. To validate their efficacy as anti-lung cancer candidates, further investigations encompassing the elucidation of the mode of action and preclinical trials are imperative.

## Figures and Tables

**Table 1:** Cytotoxic effect (IC_50_, μg/mL) of *L. pumila* extract against A549 human lung cancer cells at varying EtOH concentrations.

EtOH Concentration (%)	IC_50_ (μg/mL)
10	<100
20	<100
30	<100
40	<100
50	<100
60	<100
70	57.04 ± 10.28
80	76.94 ± 5.56
90	80.91 ± 4.31
100	84.09 ± 8.65

## Data Availability

The data presented in this study are available on reasonable request from the corresponding authors. All data generated or analyzed during this study are included in the manuscript.
